# Oncological Children and Well-Being: Occupational Performance and HRQOL Change after Fine Motor Skills Stimulation Activities

**DOI:** 10.3390/pediatric13030046

**Published:** 2021-07-05

**Authors:** Livia Taverna, Martina Bellavere, Marta Tremolada, Lietta Santinelli, Nicola Rudelli, Michele Mainardi, Graziano Onder, Maria Caterina Putti, Alessandra Biffi, Barbara Tosetto

**Affiliations:** 1 Faculty of Education, Free University of Bozen-Bolzano, 39100 Bozen, Italy; 2 Province College for Health-Care Professions Claudiana, 39100 Bozen, Italy; martina.bellavere@stud.claudiana.bz.it (M.B.); Barbara.Tosetto@claudiana.bz.it (B.T.); 3 Department of Developmental and Social Psychology, University of Padua, 35100 Padua, Italy; marta.tremolada@unipd.it; 4 CEP, Center for Occupational Therapy, 6500 Bellinzona, Switzerland; lietta@ergoterapiapediatrica.ch; 5 Learning Training Department, SUPSI, 6600 Locarno, Switzerland; Nicola.Rudelli@supsi.ch (N.R.); Michele.Mainardi@supsi.ch (M.M.); 6 Faculty of Medicine “A. Gemelli”, Catholic University of Sacred Heart, 00168 Rome, Italy; graziano.onder@unicatt.it; 7 Clinic of Pediatric Oncohematology, Department of Child and Woman Health, University of Padua, 35100 Padua, Italy; mariacaterina.putti@unipd.it (M.C.P.); alessandra.biffi@unipd.it (A.B.)

**Keywords:** occupational performance, fine motor skills, health related quality of life, leukemia children, well-being

## Abstract

Cancer children experience long periods of hospitalization, which are associated with limited performance in several developmental domains and participation restrictions in age appropriate occupations. Fine motor abilities represent building blocks in performing daily life skills and have been found to be closely connected with later academic success. Moreover, medical and psychological sequelae for cancer inpatients may result in diminished daily activities functioning, poor perceived health related quality of life (HRQOL), and increase the likelihood of long-term impairments. This study examines the variations in the occupational performance of children hospitalized for acute lymphoblastic leukemia (ALL) after their participation to a stimulation program designed to enhance fine motor skills. Parents reported significant gains in children’s motor functioning, a slight improvement in overall occupational performance related to an increase in the area of productivity and self-care, and a better quality of life perception following the stimulation activities. Feasibility of the stimulation program in a health care setting are discussed evaluating its benefits for cancer children and their families.

## 1. Introduction

The diagnosis and treatment of a malignant tumor in children represents a very stressful event for children and their families [1]. Despite the evolution of treatments and the increase in the chances of survival, children with cancer or childhood cancer survivors present numerous medical side effects and long-term outcomes that compromise their well-being and quality of life [2,3] as well as with gross and fine motor skill delays, especially if they underwent hematopoietic stem cell transplantation [4]. They also reported severe consequences on their life perceptions, in their general well-being, and in their fatigue perceptions when they were considered healed compared with healthy peers [5]. With increasing life expectancy, the experts have tried to describe and understand how the disease experience affects the future outcomes of these children in order to intervene, where possible, to facilitate the developmental trajectories of cancer survivors becoming similar to those of healthy peers. Physical and psychological suffering is not caused only by illness, but also by long months of hospitalization and therapies, with consequences that can occur both in the short- and long-term. Invasive therapies such as chemo or radiotherapy play a fundamental role in child survivorship and, however, can cause numerous side effects. These vary from patient to patient depending, for example, on general health conditions, tumor site, and type of treatment [6]. Among the short-term sequelae, we find fatigue, nausea, and vomiting, ulcers in the mucous membrane of the digestive system and mouth, taste alteration, loss of appetite, diarrhea, constipation, infections, anemia, bleeding, hair loss, and hair loss of eyelashes and eyebrows [7,8]. Furthermore, during the treatment phases, changes in strength and motor balance can occur in the child [9]. The long-term sequelae are instead identifiable in a series of physical and psychosocial disorders that depend on numerous factors: the diagnosed disease, drugs, type of treatment [3,4,5,6,7,8,9,10], age, tumor’s location, lack of stimulation, long months of hospitalization and reduced/absent interactions with friends and peers [9,11,12]. Long-term effects can involve the child’s physical, cognitive, and social developmental domain and determine motor difficulties (global and fine), attentional or executive functioning, communication, academic learning, and socialization with important consequences on the future quality of life of the child [8,13,14]. Furthermore, during the treatment phases, changes in the motor balance in strength and balance can occur in the child [9]. The long-term sequelae are instead identifiable in a series of physical and psychosocial disorders that depend on numerous factors: the diagnosed disease, drugs, type of treatment [3,4,5,6,7,8,9,10], age, tumor’s location, lack of stimulation, long months of hospitalization, and reduced/absent interactions with friends and peers [9,11,12]. Long-term effects can involve the child’s physical, cognitive, and social developmental domain and determine motor difficulties (global and fine), attentional or executive functioning, communication, academic learning, and socialization with important consequences on the future quality of life of the child [13,14].

However, recently, the literature has also focused on the coping and adaptation resources for pediatric patients that could help them to overcome the main stress of hospitalization and cancer treatments. Some studies have stressed the concept of personal growth, for example, in childhood cancer survivors assessed both by questionnaires [15] and by the narrative approach [16]. These are key elements that are an important basis for occupational intervention.

### 1.1. Playing in Hospital

Several scientific studies have focused on the positive effects induced by the introduction of playing activities in the hospital similar to those to which children would have access outside the medical context. For example, a study by He and colleagues [17] predicted that children aged 6 to 14, in addition to routine care, also received an hour of therapeutic play a few days before surgery. This included watching some videos to prepare them at the time of the operation, and some photos of the hospital and operating room and demonstrations of dolls with how anesthesia is induced. The data showed that this is a good way to reduce anxiety in children before they can intervene. This therapy is also used to give voice to the emotions that children feel during hospitalization, but cannot experience, so it is also very useful to re-elaborate with awareness what they are experiencing, given that often the frenzy of treatments and interventions can daze the patient. 

Another study [18] showed that children with leukemia established some sort of routine as their coping strategy when waiting for their treatments at the hospital, so it is important to provide children with access to similar toys and activities on a regular basis. This study stressed that children with leukemia played less than the control children, their play was characterized by repetitive themes, it was solitary, and it was less evolving and thus associated with a high anxiety intensity.

“Normalizing” the hospital aims at giving children the opportunity to perceive the medical setting as more familiar, to keep a connection to the world, and to better cope with the illness. Inpatients and their families are offered activities like cineforums, school-based exercises, and intervention programs such as pet, musical, or clown therapy to name a few. Facilities like playrooms are provided to spend some time with peers during extended hospitalization periods and to meet friends in a pleasant and stimulating environment. In general, playing for children means active experimentation that promotes learning skills, abilities to express emotions, and the opportunity to put into practice acquired skills for building competence. For this reason, parents are encouraged to support the child wherever and at any time it is possible. Any spontaneous activity that the child performs with pleasure can be defined as play. Regardless of culture and origin, children, if their health conditions allow it, find the time to play [19]. Because of the extraordinary importance of play in children’s lives, many specialists have decided to use play as a vehicle for information and communication. Playing serves as an intervention tool to allow the expression of negative emotions caused by the hospitalization experience, promotes the psychosocial well-being of young inpatients helping them to cope with the illness, and finally normalizes the medical setting, allowing the children’s developmental needs to be met and to provide experiences similar to those experienced by peers outside the hospital.

Medical play, for example, is a common technique used by health personnel to give children the opportunity to get in touch with hospital devices (needles, syringes, catheters, gauzes, plasters, thermometers, stethoscopes, etc.), in order to reduce anxiety and to cope with medical visits, therapeutic procedures, and hospitalization periods. Familiarization with medical and hospital equipment also offers children the ability to express and process their feelings, to face false beliefs, to distinguish reality from fantasy, and to better cope with illness experience, thus increasing their understanding [20,21]. Positive effects related to medical play have been variously documented and include the stress relief in dealing with surgical interventions [22,23] and to foster control over negative emotions related to one’s own hospitalization or that of a family member [24].

Therapeutic play, unlike medical play, represents a structured activity aimed at promoting psychosocial well-being [25], coping skills, and ensuring the expression of the emotions of hospitalized children. Therapeutic play includes various types of activities including expressive arts and crafts, pretend play with dolls, and puppet shows. Each therapeutic play can be planned in such a way as to ensure that interaction with health care staff is aimed at helping children express and process their emotions. The benefits deriving from therapeutic play concern the management of emotions [26], the understanding of thoughts and concerns projected in the play or in the artistic products created (drawings, masks to cover the face during therapeutic procedures) [27], and better adaptation to hospitalization [26].

Play in health care settings is used as an opportunity for normalization to face extended periods of hospitalization and to promote typical development for severely ill children. Some studies have reported that prolonged hospitalization may result in an experience of deprivation and lack of stimulation, presenting in several developmental delays in different domains such as daily living, motor, and socialization skills [28]; Ref. [4] or behavioral disturbances as expected responses to the challenging situation [29]. Playing and stimulation activities in health care settings have been introduced to reduce the gap between the life in and outside the hospital and include video games, puzzles, and reading books as well as table games. In this way, parents have the opportunity to entertain their children with stimulating activities and encourage interaction with inpatient peers in order to normalize the stay in hospital and create a bridge with life outside the health care setting. Thus, the hospital becomes a little less aseptic, closer to the developmental needs that children have despite their health conditions [30]. A more “normal” context of care, with spaces dedicated to entertainment and playing activities similar to those that the child would have access to outside the hospital, allows for a less alienating hospitalization experience and to maintain a bond with a healthy life by spending a fun times doing enjoyable activities with peers.

A prior review study on different forms of play in hospital [31] point out a lack of literature of research studies on psycho-educational play in health care settings aimed at occupational rehabilitation or intervention in developmental domains compromised by long periods of hospitalization or by therapeutic treatment. In fact, play can be used as a tool to promote the acquisition of necessary skills for later key competences and the child’s future educational goals by means of fun activities. Moreover, psycho-educational play aims at stimulating cognitive abilities, enhancing learning processes and instrumental functions while expressing, and sustaining one’s own proficiency. For this reason, the link with the normal world outside the hospital is not maintained only through the recreation of settings and opportunities resembling those experienced in the life without illness, but keeping alive the hope of existing in good health beyond illness. Thinking about the child’s future means succeeding at creating the conditions for their active participation in the social, educational, emotional life awaiting them once they are healed.

### 1.2. Hospitalization and the Risk of Occupational Deprivation

Some studies have shown that a limitation in the production and in carrying out one’s own abilities, whether temporary or lasting for extended time periods as well as the loss of an important function can lead to a state of occupational deprivation with negative consequences on later child development [32,33]. The risks associated with an impediment to achieve or even purely participating in those activities that form an integral part of a normal development path (occupational deprivation) include low self-esteem, difficulties in social adaptation, and health problems of various kinds. Being hindered to take part in daily life activities, a sudden interruption of habits and familiar routines (for example, stopping eating alone, buttoning, writing, or playing) can lead children to believe that they are incapable and incompetent, negatively influencing the perception they have of themselves and the confidence to be at the same level as their peers. Furthermore, the diagnosis and treatment of a tumor in pediatric age as well as abruptly interrupting the personal habits and autonomies of the child [34] can further involve psychosocial effects such as marginalization and isolation from the peer group [33,35]. The condition of quarantine and the hygiene requirements related to hospital care restrict interactions to adults only including health staff and specialists, close family members, thus severely impacting and limiting the child’s participation and socialization [9].

During hospitalization, the entire family nucleus is forced to adapt to the needs and times of the health facility. The new situation causes insecurities in parents with respect to their role; sometimes they feel guilty for the situation, incompetent, and incapable, caught in the grip of impotence for the severe illness of their children [36]. Feelings and emotions like the ones just described risk modifying the expectations that parents may have regarding the development and autonomy of their children, pushing them to exhibit protective care by replacing them in performing actions that they could carry out alone instead of promoting and supporting daily life skills.

### 1.3. Preschool Leukemia and Limitations in the Development of Fine Motor Skills

Preschool children with acute lymphoblastic leukemia (ALL) may experience significant changes in the development of motor skills. Neuropathies at the level of the peripheral nervous system induced by the use of treatment drugs such as vincristine may be the cause of weakness in the distal musculature [37,38], reduced muscular power in the upper and lower extremities [39,40], motor clumsiness, of poor coordination, in particular manual and bimanual [41,42], paresthesia of the fingers of hands and feet, loss of sensitivity to stimuli or altered perception of pain, temperature and proprioception, and finally loss of tendon reflexes [43]. These difficulties are reported both in the acute phase of medical treatments, and in some cases, in the less intensive phases persisting up to two years after the end of the management and care of the cancer disease [11,44,45,46].

In typically developing children, fine motor skills have been associated with mathematic and reading achievement [47,48]. In addition, the results coming from neuroimaging studies confirm the idea that there is a relationship between the first mental calculation skills and the movement of the fingers [49,50] since being able to count with hands reduces the working memory workload, allowing a higher availability of cognitive resources for learning new contents or strategies in solving problems [51]. Conversely, it could also be assumed that a poor digital dexterity could correlate with a reduced performance in problem solving or counting accuracy [52]. A possible explanation of the mechanisms underlying the relationship between fine motor skills and academic performance is that finger and hand movements allow children to create a visual correspondence one-to-one, to group objects on category basis (number, shape, size), and to develop a procedural competence at counting. Finally, digital dexterity helps children to achieve a good level of automaticity in grapho-motor skills, ensuring them the opportunity to pay more attention to the grapheme–phoneme relationship, to decode properly during reading, and to master mathematical concepts [53].

Considering the fact that the peak incidence of leukemia in infancy occurs when children are between one and four year-olds [54], the effects of reduced motor function may have long-term consequences in learning the first mathematical concepts, in the letter-sound connection, and in the emergent literacy. More studies are required on leukemic children, a population at risk of numerous developmental neurocognitive deficits, to understand the relative contribution of motor skills and visual–motor integration abilities on the acquisition of foundational academic competences [55].

Some authors have pointed out that fine motor skill difficulties are sensitive to rehabilitation [56] and can therefore be addressed with targeted psycho-educational activities.

This exploratory research aimed at observing the effects produced by psycho-educational playing activities to enhance digital dexterity and grapho-motor skills in preschool aged children hospitalized for leukemia. Moreover, the present study was designed to understand the psychological consequences of parental involvement in children’s fine motor skill stimulation to maintain positive expectations about future life, looking beyond the tumor disease.

### 1.4. Research Objectives

The primary objective of the present study was to analyze and describe the actual fine motor development of leukemia children and their perception of quality of life. A second objective of the study was to investigate possible associations between child’s socio-demographic characteristics, quality of life perception, and fine motor skills performances. Finally, we wanted to test the feasibility of the kit in a hospital setting, measuring whether it was perceived as pleasurable and stimulating, and considering its benefits for cancer children and their families.

## 2. Materials and Methods

In this study, 13 children (seven males and six females) participated, with an average age of 58.31 months (SD = 9.09; range: 44–70). Participants were treated at the Pediatric Hematology-Oncology Clinic of the Padua Hospital for acute lymphoblastic leukemia (ALL). A total of 23.1% were hospitalized (n = 3) and 63.9.0% (n = 10) came periodically to the day hospital for routine visits. Former children (30.8%) were in the consolidation phase of therapy, while the latter were in the maintenance phase (69.2%).

The children and their parents were asked to be involved in the study by the clinical psychologist of the clinic that only had knowledge of them with precedent counselling and support interviews. The psychologist also introduced the occupational therapist trainer during their stay in the hospital (day-hospital or hospitalization area) and the assessment and occupational play meetings were agreed with parents.

All parents understood and spoke the Italian language and were Caucasians except a family that was Moldovan. Parents that filled in the proxy-questionnaires consisted of seven mothers. Socio-demographic information of the pediatric patients obtained through the medical records and during the COPM interview are reported in Table 1.

### 2.1. Instruments

Data were collected using two standardized tools: the Movement Assessment Battery for Children-Second Edition (M-ABC-2) [57] and the Canadian Occupational Performance Measure (COPM) [58]. Two ad hoc questionnaires concerning satisfaction and feasibility of the kit (MoFis-I Kit) in a hospital setting were created and filled in by health care professionals and cancer children. Furthermore, children’s perceived quality of life was assessed through the administration to the parents of the PedsQL [59].

### 2.2. Movement Assessment Battery for Children-Second Edition (M-ABC-2)

The Movement Assessment Battery for Children-Second Edition (M-ABC-2) is a standardized tool used to test fine and gross motor skills with the purpose of identifying individuals with motor function impairments. Children were required to perform eight tasks grouped into three sections (manual dexterity; aiming and grasping; balance) on which they were scored and rated. The performance test M-ABC-2 covers three age bands: 3:00–4:11 years, 5:00–10:11 years, and 11:00–16:11. The present study involved the administration of the Manual Dexterity scale only, which included the following three tasks: inserting coins into a piggy bank; threading beads into a wire; and tracing a path inside margins with a pen. The M-ABC-2 provides a total score and a standardized score adjusted for age and percentile of the motor functioning of the assessed individual. The M-ABC-2 battery provides a “traffic light” system that allows for the interpretation of performance scores according to a risk categorization. Any child whose score falls at or below the 5th percentile is regarded as having a significant movement difficulty (red zone), between the 6th and 15th percentile at risk of developing motor difficulties that require to be monitored over time (amber zone), and above the 16th percentile as unlikely to have a movement difficulty (green zone).

The M-ABC-2 test requires an individual setting. The ideal assessing room should be 6 m × 4 m in size and have at least one smooth white wall (preferably without windows). Furthermore, it should be well lit and ventilated. There must be a table and two chairs available. Recommendations are provided that the table and chairs height are adjusted according to the height of the child to be evaluated. The table surface should be approximately at the level of the child’s elbow when seated. Finally, the child must be able to rest their feet firmly on the ground or on a raised platform.

The M-ABC-2 shows good psychometric proprieties with high test-retest reliability ranging across one week from 0.92 to 0.98 [60] and indicating that the Movement ABC provides stable values over a 1-wk period in all age bands. Concurrent validity estimated with the Bruininks–Oseretsk Test of Motor Proficiency (BOTMP; [61]) as the criterion measure was good (range of Pearson’s *r* values from 0.60 and 0.90) [60]. These findings have been confirmed by the study of Swee and colleagues [62], showing a correlation coefficient of 0.79 between the M-ABC-2 and the BOTMP-SF, a coefficient of 0.86 between the M-ABC-2 and the McCarron Assessment of Neuromuscolar Development (MAND; [63]) and a coefficient of 0.83 between the BOTMP-SF and the MAND.

### 2.3. Pediatric Quality of Life Inventory (PedsQL 3.0-Parent Version)

This is a paper-pencil questionnaire [59] to assess the perceived quality of life in children with cancer. Existing versions are filled in by parents of children aged 2–4 years (Toddler Module) and 5–7 years (Young Child Module). The instrument consists of 27 items grouped in different scales measuring four dimensions of health related quality of life (HRQOL): physical (pain, nausea), emotional (treatment anxiety, procedural anxiety, worry), cognitive problems (paying attention, remembering, concentration), and social (perceived physical appearance, communication). The PedsQL consists of questions designed to measure the extent of a problem in the last week using a 5-point Likert type scale (0 = never a problem; 1 = almost never a problem; 2 = sometimes a problem; 3 = often a problem; 4 = almost always a problem). The internal consistency reliability coefficients for the PedsQL Generic Core Total Scale Score of the parent proxy report was alpha = 0.93, whereas the Cancer Module score scales met or exceeded an alpha of 0.81 [64].

### 2.4. Canadian Occupational Performance Measure (COPM)

COPM is an individualized outcome measure designed to capture the change in occupational performance. It is administered as a semi-structured interview that allows respondents to identify problematic areas of occupational performance related to daily life activities of self-care, productivity, and leisure. The COPM is mainly used to identify in which areas of occupational life the individual needs to set change objectives and formulate a targeted intervention program with rehabilitation staff able to highlight any deviations from the initial assessment. The COPM is designed to target client-perceived problem skills in daily function by asking the respondent to assign a score in the self-care, productivity, and leisure domains taking into account both the perceived importance of the occupational performance areas as well as the client’s satisfaction with the present performance. The interviewee is asked to prioritize and choose up to five occupational problems by rating the importance of knowing how to perform each of the indicated activities, and how much they perceive these issues as impacting or restricting their everyday life using a scale from 1 to 10 (1 = not at all important; 10 = very important). In a second step, the five most important activities are assigned a performance score (1 = they are not able to do it; 10 = perfect performance) and one of satisfaction (1 = not at all satisfied; 10 = completely satisfied). Overall performance and satisfaction scores were calculated by dividing the sum of the scores by the number of problems. Any variances in performance and satisfaction can be detected by a second administration of the COPM after a pre-established period of intervention based on agreed goals. This post-intervention assessment enables the individual and the rehabilitation staff to have a concrete image of changes that have occurred during the therapy process, to monitor the implementation of occupational performance, and to adapt the treatment objectives. A shift of two or more points in the performance mean value is considered clinically significant [65,66]. The COPM has been validated in many populations including the Italian one. The Italian version of the COPM is a reliable instrument with good test-retest correlation coefficient after one week interval for the performance and satisfaction scores (ICC = 0.77; 95% CI: 0.52–0.89; 0.79; 95% CI: 0.56–0.90, respectively) and an overall internal consistency (COPM performance: alpha = 0.774. COPM satisfaction: alpha = 0.79) [67]. The COPM can be administered to adults and children from eight years of age [58]. In this study, the COPM was compiled by parents as first caregivers, reliable, and “expert” informants of their child’s needs, abilities, difficulties, and personality, as described in some previous works [68].

### 2.5. MoFis-I Kit Fun Questionnaire

Children were asked to rate the kit activities on a 5-level Likert scale in the following dimensions: playfulness, attractiveness, degree of interest, pleasure, and satisfaction in the execution. The questionnaire consisted of eight items, five with a closed and three with an open format.

### 2.6. MoFis-I Questionnaire

This questionnaire was designed to measure the feasibility of the MoFis-I Kit in health care settings. It consists of 13 questions to be answered by an adult for collecting information on the intrinsic characteristics of this set of implements to be assembled and worked up: low-priced, material easily available, transportable, clear instructions, pleasant, progression from simple to complex, presence of variants with different degrees of difficulty. Health professionals such as psychologists, trainees, and volunteers were asked to fill in the questionnaire answering each item on a 5-point Likert scale (1 = totally disagree; 5 = completely agree). The items concerned the feasibility of the kit, the perceived problems, the amount of time involved for psycho-educational play activities and the pleasure using it, for example: The stimulation Kit seemed to me difficult to use; It was difficult to find the material to build up by the Kit; The child was shown to be bored in doing the activities of the Kit; I would not want to use the Kit other times.

### 2.7. Place and Data Collection

Children took part in the present study after completion of the parents’ informed consent for data collection and processing, for video recording, and/or child’s hands to be photographed while carrying out a psycho-educational activity.

The M-ABC-2 and the COPM were administered in two distinct settings. Hospitalized children in the pediatric cancer ward were subjected to continuous infusion of chemotherapy and cortisone drugs by central venous catheter (CVC) and had great fatigue and difficulty in maintaining the sitting position. For these children, research instruments were administered while sitting or lying in bed. Children hosted in the day hospital performed the three manual dexterity tests of the M-ABC-2 in the play or in the school area. These children were seated during the test’s administration but the table and chair sizes were not adequate for their height.

### 2.8. Research Design and Data Analysis

The primary objective of the present study was to analyze and describe the actual fine motor development of leukemia children and their perception of quality of life. A second objective of the study was to investigate possible associations between the child’s socio-demographic characteristics, quality of life perception, and fine motor skills performances. Finally, we wanted to test the feasibility of the kit in a hospital setting, measuring if it was perceived as pleasurable and stimulating and considering its benefits for cancer children and their families.

The data analyses plan included descriptive statistics (frequencies, means, and standard deviations) to provide an overview of the results at a glance. To meet the first objective of the study, perceived quality of life of leukemia children and their fine motor skills performances were analyzed, reporting the average values alongside the standard deviations and the range scores. The second aim of the research consisted in testing possible associations between the child’s socio-demographic characteristics, quality of life perception, and fine motor skills. A Pearson product–moment correlation was run to determine the strength and direction of the linear relationship between socio-demographic variables and performance scores. Finally, the benefits obtained by administering the MoFis-I kit to leukemia children was estimated by comparing the fine motor skill scoring before (T1) and after (T2) stimulation activities to understand possible changes.

### 2.9. The MoFis-I Kit

Stimulation of fine motor skills in hospitalized children took place using the MoFis-I Kit, an intervention program designed to improve the fine and grapho-motor skills of children aged between four and six years through 15 specific activities. The psycho-educational play activities of the kit were studied in order to achieve three objectives: (a) to improve the functions of the hand, in particular manual dexterity, digital dexterity, in-hand manipulation, and visual–motor coordination, considered essential to the performance and to the participation in daily life activities and foundational for the development of grapho-motor competence; (b) to develop manipulation skills related to autonomy and learning activities; and (c) to increase manipulation strength and endurance during fine motor activities.

The 15 activities can be carried out with a set of tools and implements that are inexpensive, easy-to-find (staples, elastic bands, plasticine, plastic bottles, pencils etc.), and transportable. Instruction for assembling the related material are given in detail to facilitate the administration both by occupational therapists or by other health personnel like, for example, psychologists, nurses, educators, hospital school teachers, volunteers, and parents. All psycho-educational play activities can last even just a few minutes, are presented in order of progressive difficulty, and are attractive and funny. At each stimulation session with the kit, the child must carry out three different activities, each of which trains different fine motor skills: activities for manual dexterity, activities for digital dexterity, and activities for visual-motor coordination.

The duration of the daily stimulation depended on the health and fatigue conditions of the children. For children hospitalized in the cancer ward, the administration took place on a daily basis and mostly in bed. The duration of the intervention was a maximum of 10 min because after this time, the children’s resistance diminished significantly. It was not always possible to administer the three activities during the same session.

For the children of the day hospital, the administration was scheduled once/twice a week, based on the medical examinations the child had planned. For the children of the day hospital, the kit was performed in the area dedicated to the school, although the activities often had to be interrupted by doctors and nurses when children had to undergo routine medical visits. For these children, the intervention program had a variable duration from 10 to 50 min.

In the present study, the psycho-educational play activities were conducted mainly under the supervision of an occupational therapist and two psychologists present at the DH and in the ward.

## 3. Results

To investigate the objectives of the present study, the data collected were analyzed to describe the (1) quality of life perception of leukemia children and their actual development in the manual dexterity domain, (2) possible association between child’s characteristics, fine motor skills and quality of life, and finally (3) the feasibility of the kit in a hospital setting looking at the parents, children, and health care staff perception of its benefits and critical aspects.

### 3.1. The Perceived Quality of Life and Fine Motor Skills in Children with Leukemia

Table 2 presents the data concerning the perceived quality of life in children with leukemia.

The lowest scores were perceived in the physical and social domains as reported in Table 2, even if the standard deviations indicated a wide individual variability. The fine motor skills of cancer children are reported in Table 3 with mean standard values and range scores. Standard scaled scores of manual dexterity scale were then converted in percentiles showing that eight children were below the 50th percentile, while the remaining five were at the 50th percentile or even higher.

### 3.2. Possible Associations between the Perceived Quality of Life Constructs and Fine Motor Skills between Them and with the Other Socio-Demographic Variables of the Child

Significant negative correlation was found between age and perceived quality of life of severely ill children (r = −0.76; *p* = 0.04), showing that a lower quality of life perception was associated with a higher age, so that the older the children, they perceived a lower HRQOL. Gender, risk band related to leukemia diagnosis, and type of therapy they were going through (consolidation vs. maintenance) were not significantly associated with the perception of quality of life in any of the PedsQL scales (*p* > 0.05).

With respect to the strength and direction of the relationship between manual dexterity scores and the socio-demographic variables of the child (age, gender), no significant associations could be detected (r = ns; *p* > 0.05), while the results of non-parametric statistical analyses indicate significant differences in the average ranks of the raw scores of the non-preferred hand depending on the therapy phase (Mann–Whitney U = 5.5; *p* = 0.05). Specifically, at the beginning of the consolidation phase, children exhibit lower motor performance compared to motor functioning displayed in the maintenance (3.88 vs. 8.39), so it can be assumed that the toxicity of the initial chemotherapy negatively affected the children’s fine motor skills more in the first period of therapy than in the subsequent period.

Other significant associations were assessed between perceived quality of life and manual dexterity: there was a negative association between perceived quality of life perceived with respect to the physical area and manual dexterity relating to percentiles and standardized scores (r = −0.73, *p* = 0.05). Thus, parents declare a worse quality of life perception in the physical area in those children who performed lower in manual dexterity assessment.

### 3.3. Stimulation Activities for Inpatients: Description of Possible Benefits on Quality of Life, Motor Skills, Parental, and Care Staff Appraisal of the Kit and Pleasure Perceived by Children

With respect to psycho-educational play intervention and perceived quality of life of inpatients, the data collected did not indicate significant differences between children who performed the kit and those who did not. However, the present pilot study was conducted with a small number of children that were mainly in an intensive phase of therapy. Changes in the children’ fine motor skills pre- and po-intervention with respect to performance scores and percentiles are reported in Table 4.

Parental areas of concern are the children’s productivity, particularly academic, and self-care skills. The importance attributed to these dimensions and corresponding changes after the psycho-educational intervention are reported in the Table 5.

## 4. Discussion

This study has provided some interesting findings on leukemia children fine motor skills after using the MoFis-I Kit stimulation program, their perception of quality of life, and parent satisfaction with the psycho-education play intervention. Comparing the results of the manual dexterity raw scores at T1 with fine motor skills performed after the use of the MoFis-I Kit (T2), an average deviation could be detected, although with considerable differences at the individual level. Three of the five children who participated in the study at the first evaluation (T1) achieved performance scores placed in the green zone of the M-ABC-2 (between the 25th and 75th percentile), while a child had achieved a score below the 5th and one above the 95th percentile, indicating in one case the presence of a significant motor difficulty, and in the other of a performance skill significantly greater than that expected for age.

At post-intervention (T2), on average, children showed a deviation in the percentile of performance at M-ABC-2 of 17.6 points, passing from 47.4 to 65 (Table 4). In particular, two children achieved an improvement in the manual dexterity test. One child’s performance remained stable, while the performance of two children fell slightly. Changes in children’s fine motor competence need to be further investigated to better understand the mechanism that underlie the great variability detected. However, the MoFis-I Kit, beyond its effectiveness, obtained a very positive evaluation from the children and also from their parents.

In the semi-structured COPM interviews, parents were asked (T1) to think about their child’s daily activities and to identify the developmental areas of more concern. In addition, caregivers had to assign a score of importance to each activity. The school activities mentioned by the parents were: write your name, cut with scissors, use the glue stick, hold the pencil, and paint. In the self-care dimension, the following activities were identified: unscrew the bottle cap, fasten the shoes, close the zip, use the knife, wear the shirt, put the toothpaste on the toothbrush, and eat independently. The average importance attributed by participants to the self-care performance area (M = 7.88) was lower than that considered with concern connected with productive educational activities rated as important for future academic success (M = 8.86). At T1, parents judged the children performances at an average global level (M = 4.69). Exhibited skills related to school productivity (M = 5) were better than those related to self-care (M = 4.38). These findings correspond to globally high satisfaction levels (M = 7.52), especially in school productivity (M = 8.29) compared to self-care (M = 6.75).

Parent interviews with the COPM reported some interesting qualitative data that should be considered. The totality of the parent reports that their child was very active before the onset of the illness, but became tired, lazy, and the desire to play diminished. Moreover, it emerged in the narratives of three out of five parents that they were very satisfied with the commitment and motivation of children facing difficult activities, while the remaining two parents said that since the illness began, the children often asked for help to carry out activities that they were actually able to do. However, helping children was not a problem because this kind of support and assistance “is a way to be close to them”. Parents were likely to experience a high level of parental satisfaction in helping children despite the difficulties perceived in carrying out productivity and self-care activities. As stated by Mancaniello [36], the concern about survival can lead parents to change their expectations in taking care of the child, replacing them even in those tasks that they could perform alone. Even for parents who participated in the present study, the diagnosis and treatment of a tumor affected activities, routines, and habits related to learning and personal independence of the child, as described by Moruno and colleagues [35].

After psycho-educational stimulation activities, the parents generally reported a slight improvement in overall occupational performance (+0.91), which is associated with an increase in the area of productivity (+1.8) and self-care (+0.02). These ratings correspond to a slight improvement in satisfaction (+0.28), which relates to a slight lowering with respect to school activities (−0.09) and an increase regarding self-care skills (+0.65). These slight variations in the area of the parents’ perceived satisfaction were not surprising since, on average, there were not even high deviations in children’s manual dexterity performances between T1 and T2.

The COPM is reported to be sensitive to changes in individual occupational performance [69,70]. The manual of the COPM states that the cut-off to detect a significant improvement over time is equal to 2 or more points of deviation from the initial mean ratings. A minimal clinically important difference of 1.4 points has been suggested for the COPM performance score based on a study with adults with specific health problems [70]. Participants of the present study did not show from baseline to second assessment time point a mean difference greater than 2 points in the productivity domain nor in the self-care dimension. The totality of the parents during the interview with T2 claimed to have noticed positive changes in the general well-being of their children. Despite the fatigue and side effects of the therapies, children often asked to play and build the kit objects together. For example, a parent during the COPM interview stated:

“[…] at times when he doesn’t sleep, he seems to me more smiling: he laughs, jokes and is amused when he takes part in the Kit games. Despite the fatigue, after the activities she is satisfied and in a good mood. For a moment it seems to me to go back, when the disease was not there yet!”.

A parent of another child made it clear that:

“[…] compared to the past few weeks I see him more active and smiling. When this is the case, I forget for a moment of the place where we are”;

The need for normality also emerged in a third interview:

“Since my son knows that in Day Hospital he can play with the Kit, he is happy and peaceful, I see him laughing often, despite having to undergo visits and blood sampling that hurt him and scare him”.

The statements reported here attest that the kit promotes a change in perspective of the parents. The introduction of playful psycho-educational activities to stimulate fine and graphomotor skills gave parents the pleasure of seeing their children in action and facilitated a dyadic relationship focused on play rather than on health conditions. The interviews with parents of leukemia children confirm the thesis of Polatajko and Mandich [47], according to which being able to participate in occupational activities allows young inpatients to learn and develop physical, cognitive, social, and emotional skills through action, to pose new challenges, to strengthen their self-esteem and self-confidence, a sense of belonging and social participation, which are fundamental issues for present and future health and well-being. On the other hand, the parents’ statements clearly bring out a need for normality that is expressed in the necessity of seeing and fostering the healthy part of their children even in a setting of care and in the requirement of dealing with the skills and competences demanded for re-entering in the world of peers. The concern for life outside and beyond the disease emerged repeatedly in the interviews with parents who participated in the present study and it has become tangible in the repeated requests to health personnel to have at their disposal different materials to enhance fine motor skills. In addition, one parent expressed concern throughout the COPM interview that their child, unable to go to school as their peers, would have encountered difficulties in learning to write.

Finally, a third parent claims that, after the onset of the disease, a pneumonia and a meningitis, “the child spent three months in bed, never being stimulated”. Statements such as the latter report attention to the perceived need on the part of families to keep children occupied, and to feel a “space of normality” in which skills and competences are still cared for.

The totality of the children (five out of five) states that playing with the kit was funny and showed an appreciation of the psycho-educational activities. Moreover, they declared that the difficulty level was appropriate to their abilities. Four children out of five stated that they wanted to continue playing, and that they managed to make all the games offered in the kit. So far, we can conclude that for the children as well as for their parents, the MoFis-I Kit represented a globally positive experience.

According to the care staff interviewed, the kit has many advantages. First, the materials are cheap and easy to find and the fact that it is contained in a box makes it easily transportable. The psycho-educational activities are mostly simple to administer but sometimes instructions have been considered as not adequate. Furthermore, administration time for some activities has not always been satisfactory because more time would be required to complete the games. Care staff respondents reported that the moments during which the kit had been used were pleasant and that it was easy to involve children in the psycho-educational activities because they were intuitive and enjoyable.

Results of this exploratory study do not confirm the previous research, which detects fine motor difficulties after the re-induction phase [45] as well as in the maintenance phase [46], related to the age of the child at the time of diagnosis and the duration of hospitalization [46]. However, we must take into account some aspects that affect the reliability of our data collection.

First of all, the test administration conditions may have altered the fine motor performance of our participants. Positioning in bed or tables that are not suitable for the height of children may have had an influence on their performance. Smith-Zuzovsky and Exner [71] showed that children aged between 6 and 7.6 without motor difficulties who had an optimal sitting position (size of tables and chairs corresponding to the height of the child, feet on the ground, knees at 90°, back against the backrest) obtained the best results in the digital dexterity tasks of the In-hand Manipulation Test-Quality section test (IMT-Q) compared to children who sat on a too large chair, not allowing them to obtain a stable and optimal position.

Furthermore, the secondary effects of cancer treatments in the young inpatients involved a great fatigue affecting their performance, which fluctuated according to the time of the day chosen for fine motor skill assessments. Furthermore, the percentile score of the Manual Dexterity Scale of the M-ABC-2 was based on only three tasks so that the failure of a single task influenced the global test result compared with the norm values. Finally, the distance between the pre- and post-intervention administration did not respect the minimum distance of six months recommended to avoid a bias due to the learning effect.

Despite the limitations outlined above, this study also presents novel elements and provides some indications on how to support leukemia children and their families. The introduction of the kit in a hospital setting clearly shows that offering stimulating psycho-educational playing activities helps young inpatients and their parents to face the difficult time of hospitalization during cancer disease and treatments. Interviews with parents report their concern regarding the future life of children, the need to support skills that are required when coming back to school, and the expressed desire to see young patients happy and active even during illness. Several studies have shown how important it is to intervene on the family as a system to support the resilience of cancer children [72]. In this sense, the kit responds to a deep psychological need that can activate resources and positive energies. The MoFis-I Kit appears to be, according to parents and staff care appraisals, a positive and stimulating tool to be adopted in contexts of health care such as oncological wards because of its ease of use and is pleasant for children.

Finally, the occupational related interventions, as suggested in previous studies [4,12], can reduce the psychosocial impact during and after chemotherapy, promoting social inclusion and supporting children and their patients’ families. For example, a pilot study demonstrated that home-exercise intervention during ALL maintenance therapy was feasible and had promise for efficacy [73]. Moreover, a recent study of Dehghan and colleagues demonstrated that children with cancer (8–18 years old) had a variety of problems in three occupational areas (i.e., self-care, productivity, and leisure), confirming the results of our exploratory research [74]. Fine motor skills are sensitive to rehabilitation and targeted intervention [56,75,76].

Berninger and Fuller [77], for example, suggest that handwriting may be particularly challenging for students who lack foundational skills in writing. The transition from kindergarten to first grade is an important period to develop and practice fine motor skills. In an observational study on 4 year-old children, Marr and colleagues [78] reported that kindergarteners spent approximately half of their school day engaged in fine motor tasks (range of 36–66%). About 20% of these activities were paper and pencil activities for either play or learning (writing or coloring with a pencil, crayon, or marker, or painting with a paintbrush). Two years later, children in second grade were found to spend as much as 30–60% of their day participating in an activity that required fine motor skills, of which 85% involved paper and pencil tasks [79]. On the basis of the above-mentioned studies, it could be hypothesized that children beginning elementary school with important delays in fine and gross motor domains could be more at risk for academic achievement. Moreover, longer hospitalizations and necessary treatments contribute to limiting the discovering of motor functioning at this age stage, forcing the young patients to stay in bed, and to avoid social and physical contacts due to their immunocompromised status. For these reasons, the MoFis-I Kit could contribute to help children maintain a level of occupational activity during long hospitalization periods. Psycho-educational play stimulates the patients’ fine motor skills and occupational performance related to the skills required in a healthy life. Furthermore, this type of stimulation allows parents to perceive their children as most active and support their resilience to the disease. Finally, the kit appears to be an easy to use, pleasant, transportable tool, suitable for the hospital setting, and enjoyable for children. Tools like the MoFis-I Kit should be introduced in hospitals to offer children with cancer the chance to support their motor development, psychological well-being, and perception of quality of life during long hospitalization periods, caring for those skills required beyond the illness.

## Figures and Tables

**Table 1:** Sociodemographic information of the participants to the present study.

	Mean	SD
Age in months (range 44–70)	58.30	9.09
	**Frequency**	**Percentage**
Assessment time	Acute period = 4	30.8
	Maintenance period = 9	69.2
Age groups	44–53 months = 6	46.2
	62–70 months = 7	53.8
Gender	6 females	46.2
	7 males	53.8
Leukemia Risk band	HR = 7	53.8
	SR = 6	46.2

**Table 2:** Means, standard deviations, and score ranges of the PedsQL in children with cancer (T1).

	Mean	SD	Range
PedsQL Total	72.97	14.88	56.48–97
PesQL Physical	69.13	16.37	50–96.88
PedsQL Emotional	77.67	18.34	53.13–100
PedsQL School	74.96	13.95	60–100
PedsQL Social	69.34	21.40	37.50–100

**Table 3:** Manual dexterity standard scores of cancer children at the M-ABC-2 (T1).

	Mean	SD	Range
MD1 preferred hand	8.58	4.27	1–15
MD1 other hand	7.69	3.19	1–13
DM2	9.31	3.27	4–14
MD3	8.46	3.12	3–12
MD total score	26.42	6.73	16–38
MD total score standard	8.92	3.09	5–15
MD total percentile	39.23	30.39	5–95

**Table 4:** Descriptive analysis of variances in performance scores of ALL children in M-ABC-2 pre- and post-intervention.

	T1	T2	Change
Manual Dexterity			
Mean	28.4	31.8	+3.4
Median	28	32	+4
Range	18–38	11	
Percentile			
Mean	47.4	65	+17.6
Median	37	63	+26
Range	5–95	54	

**Table 5:** Mean productivity and self-care importance scores and mean differences between pre- and post-psycho-educational intervention at the COPM.

Activity	Importance	COPM P T2	COPM P T1	M diff. T2-T1	COPM S T2	COPM S T1	M diff. T2-T1
Productivity	8.86	6.8	5	+1.8	8.2	8.29	−0.09
Self-care	7.88	4.4	4.38	+0.02	7.4	6.75	+0.65

*Note*: COPM P = Performance; COPM S = Satisfaction; T1 = Pre-intervention; T2 = Post-intervention.

## Data Availability

The data presented in this study are available on request from the corresponding author. The data are not publicly available due to ethics restrictions.
